# Encapsulation of *Bifidobacterium animalis* subsp. *lactis* Through Emulsification Coupled with External Gelation for the Development of Synbiotic Systems

**DOI:** 10.1007/s12602-022-09993-7

**Published:** 2022-09-29

**Authors:** Georgia Frakolaki, Virginia Giannou, Constantina Tzia

**Affiliations:** Laboratory of Food Chemistry and Technology, School of Chemical Engineering, National Technical University of Athens, Athens, Greece

**Keywords:** Emulsification, Encapsulated BB-12, Survival rate, In vitro gastrointestinal simulation, Surface morphology (SEM)

## Abstract

Aim of this work was the development of integrated and complex encapsulating systems that will provide more efficient protection to the probiotic strain *Bifidobacterium animalis* subsp. *lactis* (BB-12) in comparison to the conventional plain alginate beads. Within the scope of this study, the encapsulation of BB-12 through emulsification followed by external gelation was performed. For this purpose, a variety of alginate-based blends, composed of conventional and novel materials, were used. The results demonstrated that alginate beads incorporating 1% carrageenan or 2% nanocrystalline cellulose provided great protection to the viability of the probiotic bacteria during refrigerated storage (survival rates of 50.3% and 51.1%, respectively), as well as in vitro simulation of the gastrointestinal tract (survival rates of 38.7 and 42.0%, respectively). The incorporation of glycerol into the formulation of the beads improved the protective efficiency of the beads to the BB-12 cells during frozen storage, increasing significantly their viability compared to the plain alginate beads. Beads made of milk, alginate 1%, glucose 5%, and inulin 2% provided the best results in all cases. The microstructure of beads was assessed through SEM analysis and showed absence of free bacteria on the surface of the produced beads. Consequently, the encapsulation of BB-12 through emulsification in a complex encapsulating system was proved successful and effective.

## Introduction

Probiotic bacteria, specifically bifidobacteria, constitute a significant part of the human gut microflora. Additionally, they have been widely incorporated into various fermented foods and dairy products [1]. The consumption of bifidobacteria has been associated with certain health benefits conferred to the human host, including reduction of serum cholesterol levels, enhancement of immune function, diarrhea alleviation, decrease of lactose intolerance, modulation of the gut microflora, and allergy alleviation [2]. However, in order to exert their health benefits, probiotic bacteria should be able to survive during food processing and storage, as well as under the harsh conditions of the gastrointestinal (GI) system, in order to successfully colonize the colon [3]. Due to their high sensitivity to various environmental factors, such as heat, high acidity, oxidative stress, freezing, and moisture, probiotic bacteria are prone to cell wall deterioration, lipid oxidation, or undesirable alterations of the cell membrane [4]. Therefore, the protection of probiotics is necessary and for this purpose their encapsulation in suitable carriers has been proposed. Various methods are reported for the encapsulation of probiotic bacteria, including extrusion, emulsification, coacervation, spray-drying, or freeze-drying [5]. Εncapsulation of probiotic cells has been studied mainly through the application of the spray drying technology, using various materials such as alginate and chitosan [6], maltodextrin along with whey protein concentrate, skim milk powder or sodium caseinate, and/or trehalose or d-glycose [7] or even hydrolyzed black waxy rice flour [8]. In contrast, milder techniques such as emulsification have not been extensively examined.

Rodrigues et al. [9] encapsulated probiotic bacteria in alginate beads through the extrusion method and studied their viability during storage at 5 °C. Encapsulation had a beneficial effect, whereas the double coating with chitosan or dextran sulfate did not significantly enhance the viability of the cells. The extrusion method has, also, been extensively examined in a previous study [10], using a variety of encapsulating blends and providing satisfactory results in the protection of probiotic cells during storage or in vitro simulation of the GI tract. In the current study, the application of the emulsification method for the encapsulation of the probiotic strain, *Bifidobacterium animalis* subsp. *lactis* (BB-12), was selected to be examined, as it involves mild conditions and presents low cost and high cellular retention [1].

The emulsification technique includes the dispersion of probiotic cells in a water-based polymer suspension (discontinuous phase), which is then added in an appropriate amount of oil (continuous phase) in order to form a water-in-oil emulsion; it is substantially based on the association and interactions between the discontinuous and continuous phase. The subsequent addition of a calcium chloride solution leads to the insolubilization of the water-soluble polymer and the formation of gels beads within the oil phase, thus encapsulating the probiotic bacteria. The beads produced by emulsification may be of a wide range of shapes and sizes, whereas their diameter may be sufficiently small, even below 300 μm [5]. This technique also presents the potential for large-scale production, due to bulk beads’ formation in short time [11].

Biocompatible and non-toxic materials are investigated for the incorporation of encapsulated products in food matrices [12]. In particular, for the encapsulation through emulsification, the use of sodium alginate as encapsulating agent has already been reported [1], as it is inexpensive, nontoxic, and compatible with most other materials [13]. Moreover, alginate is widely used as an encapsulating material due to its network developing ability under mild conditions [14]. However, the application of alginate alone is not effective enough, due to its instability in the presence of Ca^2+^ chelating agents and monovalent ions or harsh conditions [14]. In order to improve the chemical and mechanical stability of alginate beads, the combination of alginate with other polymers has been proposed, such as gellan gum [15] or corn starch [16]. However, research on the combination of sodium alginate with a variety of materials for the reinforcement of the beads is still limited. Emulsification can be further combined with spray-drying [17] or freeze-drying [18, 19], since extension of probiotics’ shelf life can be achieved by reducing the moisture levels [16]. Intense drying conditions, however, have a detrimental effect on probiotics’ viability. Thus, milder approaches are recommended so as to improve the existing drying systems. The incorporation of prebiotic substances (inulin or Hi-maize starch) into the alginate systems in order to stimulate the growth and activity of probiotic bacteria has also been studied [20–23].

In this work, the elaboration of integrated and complex encapsulating systems consisting of sodium alginate, other hydrocolloid materials (xanthan gum, carrageenan, pectin, and cellulose nanocrystalline-CNC), milk and/or milk proteins, glucose, and prebiotics (inulin) is investigated. Additionally, the incorporation of cryoprotectants (glycerol) or oxygen scavengers (l-cysteine-HCl) is examined. The occurring blends are evaluated and compared regarding their effectiveness, in terms of protecting BB-12 cells during refrigerated or frozen storage as well as their transition through a simulated gastrointestinal system.

## Materials and Methods

### Materials

The probiotic culture of *Bifidobacterium animalis* subsp. *lactis* BB-12 was obtained from Chr. Hansen (Hoersholm, Denmark). Sodium alginate, calcium chloride, xanthan gum, κ-carrageenan, pectin, inulin, glucose, and Tween 80 were provided by Sigma-Aldrich Chemie GmbH (Taufkirchen, Germany); glycerol was purchased from Lach-Ner (Brno, Czech Republic); and nanocrystalline cellulose (CNC) from CelluForce (Montreal, Canada). Olive pomace oil was kindly provided by MINERVA S.A. (Athens, Greece) and sweet whey with the following specifications: humidity 1%, fat content 1%, protein content 10.2%, lactose (hydrated) 75%, ash content 7.3%, was kindly provided by ION S.A. (Piraeus, Greece). Whole-fat milk was obtained from the local market. The materials for the microbiological analyses, such as MRS agar, Ringer’s solution, citric acid, and disodium phosphate, were acquired from Merck (Taufkirchen, Germany), whereas l-cysteine-HCl, neomycine sulfate, nalidixic acid, lithium chloride, and paromomycine sulfate by Thermo Fischer Scientific (MA, USA). Pepsin, pancreatin, and bile extract were obtained from Acros Organics (New Jersey, USA).

### Encapsulation of BB-12 Cells Through Emulsification

The solutions of the various encapsulating blends were prepared according to the formulations presented in Table 1, sterilized at 121 °C for 15 min, and cooled at 35–40 °C prior to the encapsulation procedure. The CaCl_2_ solution was also sterilized at 121 °C for 15 min and cooled at ambient temperature. The glassware required for the encapsulation procedure was also sterilized and cooled under the same conditions. The probiotic strain BB-12 was incorporated at a concentration of 5% w/v into the encapsulating blend in order to form the aqueous phase. The oil phase was prepared by mixing Tween 80 (1.5% w/v) with olive pomace oil. Subsequently, the aqueous phase was dispersed into the oil phase at a ratio of 1:3. For the formation of the emulsion, the mixture was homogenized by a high-speed homogenizer (CAT Unidrive 1000; CAT Scientific, Paso Robles, CA, USA) at 1200 rpm for 5 min. Subsequently, a 0.5 M CaCl_2_ solution was slowly titrated to the emulsion under magnetic stirring, in order to cross-link the water-soluble polymers and form particles within the oil phase. The formed beads were allowed to harden/cross-link for 30 min under magnetic stirring at room temperature and were then harvested by centrifugation (10,000 rpm—approx. 11,000 g—for 15 min), washed with sterilized distilled water, and stored in sterile conical tubes at 4 °C. The above-described procedure was performed under aseptic conditions.

**Table 1:** Composition and nomenclature of the blends used for the encapsulation of BB-12

**Composition**	**Nomenclature**
Sodium alginate 2% (w/v), Glucose 5% (w/v)	A/EM
Sodium alginate 2% (w/v), Glucose 5% (w/v), Inulin 2% (w/v)	AI/EM
Sodium alginate 2% (w/v), Glucose 5% (w/v), Xanthan gum 0.5% (w/v)	AX/EM
Sodium alginate 2% (w/v), Glucose 5% (w/v), Xanthan gum 0.5% (w/v), Inulin 2% (w/v)	AXI/EM
Sodium alginate 2% (w/v), Glucose 5% (w/v), Glycerol 11% (w/v)	AGl/EM
Sodium alginate 2% (w/v), Glucose 5% (w/v), Glycerol 11% (w/v), Inulin 2% (w/v)	AGlI/EM
Sodium alginate 2% (w/v), Glucose 5% (w/v), Carrageenan 1% (w/v)	AC/EM
Sodium alginate 2% (w/v), Glucose 5% (w/v), Carrageenan 1% (w/v), Inulin 2% (w/v)	ACI/EM
Sodium alginate 2% (w/v), Glucose 5% (w/v), Carrageenan 1% (w/v), L-cysteine-HCl 0.5% (w/v)	ACL-cys/EM
Sodium alginate 2% (w/v), Glucose 5% (w/v), CNC 2% (w/v)	ACNC/EM
Sodium alginate 2% (w/v), Glucose 5% (w/v), CNC 2% (w/v), Inulin 2% (w/v)	ACNCI/EM
Sodium alginate 2% (w/v), Glucose 5% (w/v), CNC 2% (w/v), L-cysteine-HCl 0.5% (w/v)	ACNCL-cys/EM
Sodium alginate 3% (w/v), Glucose 5% (w/v), Whey 1% (w/v), Pectin 1% (w/v)	AWP/EM
Sodium alginate 3% (w/v), Glucose 5% (w/v), Whey 1% (w/v), Pectin 1% (w/v), Inulin 2% (w/v)	AWPI/EM
Milk (used instead of water), Sodium alginate 1% (w/v), Glucose 5% (w/v)	AM/EM
Milk (used instead of water), Sodium alginate 1% (w/v), Glucose 5% (w/v), Inulin 2% (w/v)	AMI/EM
Milk (used instead of water), Sodium alginate 1% (w/v), Glucose 5% (w/v), L-cysteine-HCl 0.5% (w/v)	AML-cys/EM

### Measurements

#### Viable Count of the Probiotic Cells

In order to evaluate the survival of BB-12 during the encapsulation process, cell counts were determined after the emulsification. Cell counts were obtained by determining the number of cfu in 1 g of beads. For this purpose, 1 g of the produced beads was suspended in 9 mL of citrate–phosphate buffer (pH 7.0) and disaggregated in a stomacher until cells were completely released. Samples were 10 times serially diluted in Ringer’s solution and plated between 2 layers of modified MRS agar with 0.3% v/v l-cysteine hydrochloride and 0.5% v/v NNLP (Neomycine sulfate, Nalidixic acid, Lithium chloride and Paromomycine sulfate). After 72 h of anaerobic incubation at 37 °C, cell counts were determined and expressed as log cfu g^−1^.

#### Determination of the Encapsulation Yield of the Beads

The encapsulation yield (EY) is a measurement combining the entrapment efficacy and the survival of viable cells during encapsulation, and was calculated as follows [24]:

$$\mathrm{EY=(log N/ logN_{o})}\times100$$

where N is the number of the viable encapsulated cells released from the beads and N_o_ is the theoretical number of cells estimated according to the number of probiotic cells added prior to the encapsulation.

#### Survival of Encapsulated BB-12 During Storage

The beads containing the encapsulated BB-12 cells were stored at 4 °C and –18 °C for a 30-day period. Their survival was evaluated at 10-days intervals through microbiological analysis, as described in “Viable Count of the Probiotic Cells” section.

#### Survival of Encapsulated BB-12 Under simulated Gastrointestinal Conditions

This analysis was conducted according to our previous study [10] based on the research of Holkem et al. [25]. Then, 0.5 g of beads were added in 5 mL of simulated gastric fluid (SGF) (0.025 g pepsin mL^−1^ in HCl 0.1 N, pH 2.0) and incubated at 37 °C for 90 min. Subsequently, 2.5 mL of simulated intestinal fluid (SIF) (12 g L^−1^ of bile extracts and 2 g L^−1^ of pancreatin in 0.1 M NaHCO_3_, pH 5.0) was added to the incubated mixture that was adjusted to pH 5.0 and incubated at 37 °C for 30 min. Finally, the pH was adjusted to 6.5 and the incubation was continued for another 90 min. Bacterial enumeration was executed as described in “Viable Count of the Probiotic Cells” section.

#### Surface Morphology and Bead Size Determination

A scanning electron microscope (QUANTA 200, Thermo Fisher Scientific, USA) at an accelerating voltage of 25 kV was used to characterize the shape and the external surface of the beads produced according to the various formulations. The beads were freeze-dried, fixed in stubs with double-sided copper tape, and coated with a thin gold layer (180 s at a current of 40 mA) using a Baltzer evaporator (Baltec SCD50, Liechtenstein, Austria) before being observed in the microscope.

#### Statistical Analysis

All experimental results were submitted to analysis of variance (ANOVA) using the Statistica Software version 12 (Statsoft Inc., Tulsa, OK, USA). When significant differences were observed, the Duncan’s test was applied in order to compare means at a 5% significance level. The experiments were performed in triplicate, the measurements were replicated 3 times, and their mean values are presented.

## Results and Discussion

### Influence of the Various Encapsulating Blends on the Encapsulation Yield

A variety of encapsulating blends were used for the encapsulation of the probiotic strain BB-12 through emulsification, as described in “Encapsulation of BB-12 Cells Through Emulsification” section. The blends were selected in order to reinforce the alginate beads by developing a denser or more stable grid. The conventional or novel materials used for this purpose were selected depending either on their gelation properties or their ability to provide BB-12 cells a nutritive, cryo-protective, or more anoxic environment. The entrapment of a satisfactory number of live probiotic bacteria inside the beads is of high importance as it is directly related to the number of viable probiotic cells at the end of the storage period or after their transit through the gastrointestinal (GI) system. The emulsification process applied in this study obtained high encapsulation yield (EY) values, indicating high survival of cells under the specific processing conditions, as shown in Table 2. According to the EY results, although alginate can provide a satisfactory level of protection, when combined with other hydrocolloid materials it can be much more effective, leading to greater EY values. Hence, when plain alginate (A) or alginate with inulin (AI) was used, the lowest EY values were obtained (86.9–84.7%). Similar results were found by Song et al. [11] who achieved EY of about 77–80% by encapsulating yeast cells in alginate through emulsification, and coating them with chitosan.

**Table 2:** EY values achieved for each of the encapsulating blends used. Samples’ nomenclature is defined in Table 1

***Encapsulating blend***	***EY (%)***	***Encapsulating blend***	***EY (%)***	***Encapsulating blend***	***EY (%)***
***A***	86.89 ± 1.53^aB^	***AI***	84.74 ± 1.95^aA^	***ACL-cys***	97.19 ± 1.99^dC^
***AX***	87.06 ± 2.23^aB^	***AXI***	85.20 ± 1.75^aA^		
***AWP***	87.37 ± 3.21^aB^	***AWPI***	84.18 ± 2.22^aA^	***ACNCL-cys***	97.07 ± 2.05^cC^
***AGl***	92.60 ± 1.05^bB^	***AGlI***	90.05 ± 1.37^bA^		
***AC***	98.85 ± 1.27^ dB^	***ACI***	96.68 ± 1.74^dA^	***AML-cys***	99.74 ± 1.13^eC^
***ACNC***	96.17 ± 3.38^cB^	***ACNCI***	95.28 ± 2.53^cA^		
***AM***	99.23 ± 1.14^eB^	***AMI***	98.21 ± 1.06^eA^		

On the other hand, the incorporation of xanthan gum (AX), κ-carrageenan (AC), and nanocrystalline cellulose (ACNC) in the alginate beads, as well as whey in combination with pectin (AWP) provided significantly (*p* < 0.05) higher EY values, up to 98.9%. The utilization of milk for the development of milk-alginate beads (AM) provided the most satisfactory EY values, reaching up to 99.2%. The effectiveness of the combination of these materials is attributed to the development of a denser and more stable grid that is able to retain and protect a greater number of probiotic cells. The above results come in agreement with other researchers that attempted the reinforcement of the alginate system with other materials, such as starch. Martin et al. [16] examined the development of alginate and alginate-starch beads by applying the same technique and also achieved increased EY values ranging between 74.4 and 97.3% by incorporating starch. Similarly, Khosravi Zanjani et al. [26] encapsulated the probiotic strains *Lactobacillus* (*L.*) *casei* and *Bifidobacterium* (*B*.) *bifidum* in alginate-gelatinized starch beads, with or without chitosan coating, achieving very high EY values of 96.4–98.1%. Furthermore, in the current study, the additional incorporation of inulin or l-cysteine-HCl did not significantly enhance the encapsulation efficiency.

Values shown are means ± standard deviations (*n* = 3). Values with different superscripts are significantly different. Small lettered superscripts are used to differentiate values between rows (different encapsulating agents), while capital lettered superscripts to differentiate values between columns (different additives).

### Stability of Encapsulated BB-12 Under Refrigerated and Frozen Storage

**Fig. 1:**
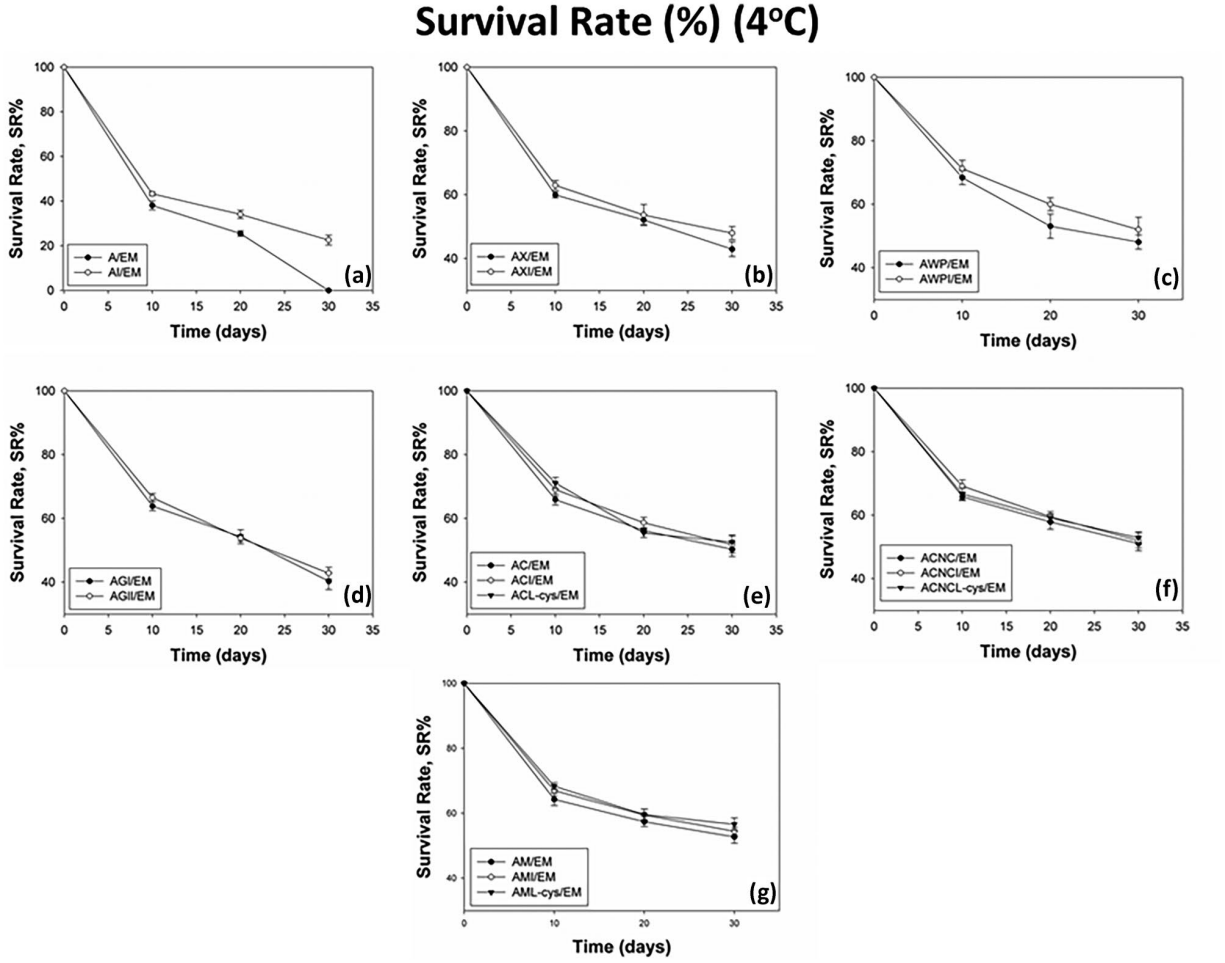
Survival rates of viable BB-12 cells during storage at 4 °C for 30 days. * Values shown are means ± standard deviations (*n* = 3). ** Sample codes: A/EM: alginate, AI/EM: alginate-inulin, AX/EM: alginate-xanthan, AXI/EM: alginate-xanthan-inulin, AWP/EM: alginate-whey-pectin, AWPI/EM: alginate-whey-pectin-inulin, AGl/EM: alginate-glycerol, AGlI/EM: alginate-glycerol-inulin, AC/EM: alginate-carrageenan, ACI/EM: alginate-carrageenan-inulin, ACL-cys/EM: alginate-carrageenan-l-cysteine-HCl, ACNC/EM: alginate-CNC, ACNCI/EM: alginate-CNC-inulin, ACNCL-cys/EM: alginate-CNC-l-cysteine-HCl, AM/EM: alginate-milk, AMI/EM: alginate-milk-inulin, AML-cys/EM: alginate-milk-l-cysteine-HCl

**Fig. 2:**
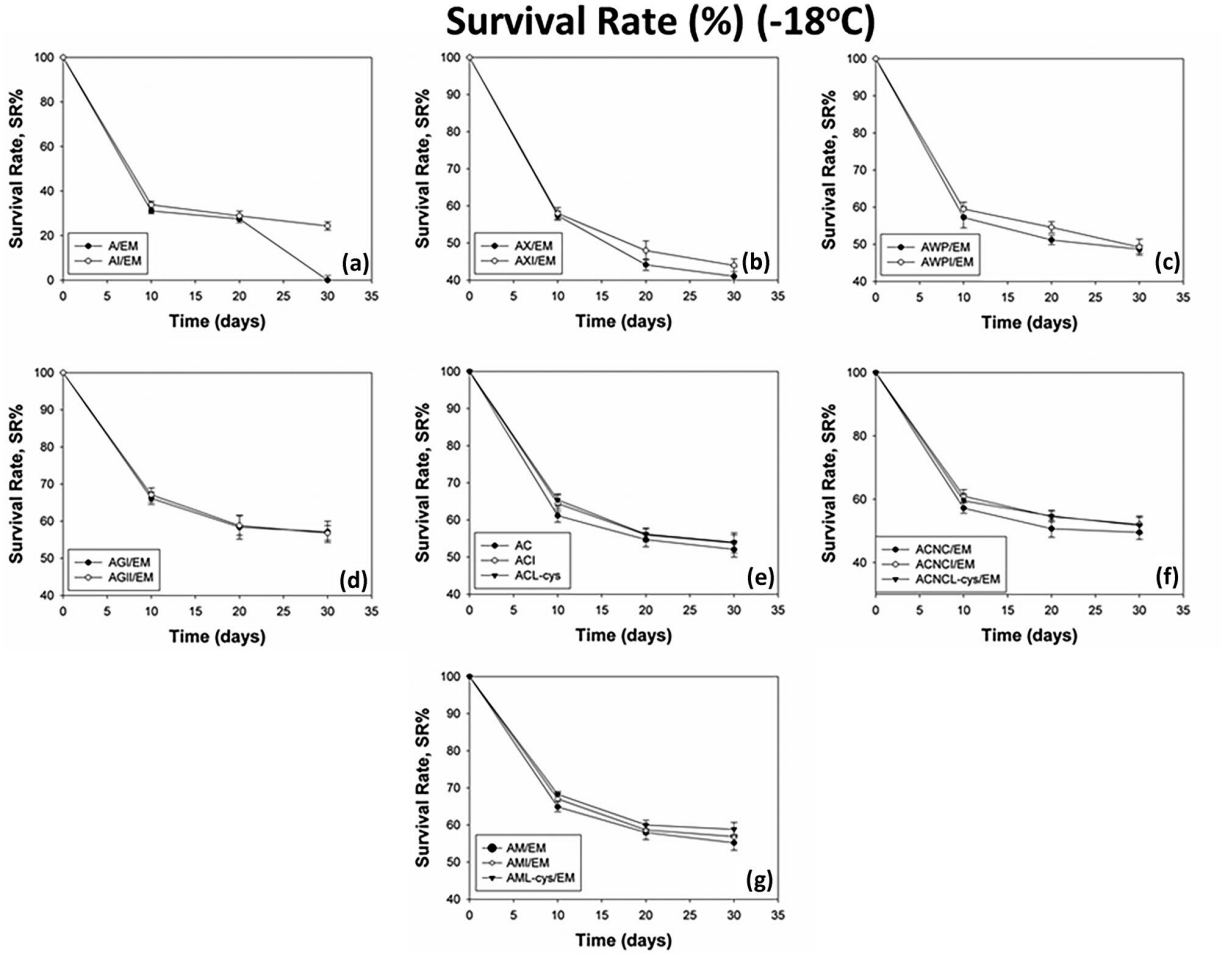
Survival rates of viable BB-12 cells during storage at -18ºC for 30 days. * Values shown are means ± standard deviations (*n* = 3). ** Sample codes: A/EM: alginate, AI/EM: alginate-inulin, AX/EM: alginate-xanthan, AXI/EM: alginate-xanthan-inulin, AWP/EM: alginate-whey-pectin, AWPI/EM: alginate-whey-pectin-inulin, AGl/EM: alginate-glycerol, AGlI/EM: alginate-glycerol-inulin, AC/EM: alginate-carrageenan, ACI/EM: alginate-carrageenan-inulin, ACL-cys/EM: alginate-carrageenan-l-cysteine-HCl, ACNC/EM: alginate-CNC, ACNCI/EM: alginate-CNC-inulin, ACNCL-cys/EM: alginate-CNC-l-cysteine-HCl, AM/EM: alginate-milk, AMI/EM: alginate-milk-inulin, AML-cys/EM: alginate-milk-l-cysteine-HCl

Refrigerated and frozen storage are widely used for food preservation in order to extend the shelf life by delaying the growth of microorganisms and the chemical reactions that cause spoilage or quality degradation in food products. Thus, the viability of encapsulated BB-12 cells was investigated under these two storage conditions in order to resolve the potential of their incorporation into a variety of food products. The encapsulating blends examined were those that led to satisfactory EY values, as described in “Influence of the Various Encapsulating Blends on the Encapsulation Yield” section and, thus, their ability to maintain the viability of BB-12 under frozen or refrigerated storage was investigated. The survival rates of the encapsulated BB-12 cells over storage at 4 °C or –18 °C was monitored at 10-day intervals during a 30-day period and the results are illustrated in Figs. 1 and 2 respectively.

#### Survival Rates of Encapsulated BB-12 Under Refrigerated Storage (4 °C)

The BB-12 cells encapsulated in alginate alone or in alginate with inulin suffered significant reductions in their viability (Fig. 1a). The survival rates were decreased at 38.1–43.3% during the first 10 days of storage, whereas no viable cells were detected in alginate beads by the end of the storage. The incorporation of inulin in the alginate blend slightly enhanced the bacterial viability, leading to the survival of 22.5% of their initial load by the end of the storage. Alginate on its own cannot provide efficient protection to the BB-12 cells, therefore its combination with the examined conventional polymer materials, such as xanthan gum (AX) and κ-carrageenan (AC) was essential. This approach was successful, as the survival rates of the encapsulated cells were significantly increased (*p* < 0.05), exceeding 42.9% and 50.3%, respectively, by the end of the 30-day storage (Fig. 1b, e). The optimal protection was achieved in the case of AM beads, as the survival rates were above 52.7%, thus indicating that this formulation was effective in protecting BB-12 cells during refrigerated storage from external factors, such as moisture or oxygen. Whey is also another material widely used for the encapsulation of probiotic strains through spray-drying due to its protective properties [27–29]. In the current study, it was combined with pectin and alginate (AWP), providing survival rates up to 48.1% by the end of storage at 4 °C (Fig. 1c). It must be taken into account that the heat denaturation of whey proteins occurring during sterilization may impact their emulsification properties and their encapsulation ability. The incorporation of glycerol (AGl) also provided increased protection to the BB-12 cells during refrigerated storage (Fig. 1d). Additionally, it must be noted that the utilization of the novel nanomaterial CNC for the development of probiotic beads (ACNC) contributed to the increase of the survival rates of BB-12 reaching 51.1% at the end of the 30-day storage period, indicating its reinforcing properties when combined with sodium alginate (Fig. 1f). Furthermore, the utilization of the prebiotic inulin significantly enhanced (*p* < 0.05) the BB-12 cells’ survival in all cases, leading to 0.9–22.5% higher survival rates compared to samples containing alginate only. Our results are in agreement with other researchers that observed improved viability during storage by encapsulating various probiotic strains in particles containing inulin [30, 31].

Although refrigerated storage is commonly recommended in order to maintain cells’ viability, and encapsulation may provide the anaerobic conditions necessary for the oxygen-sensitive BB-12 strain, a significant decrease of the bacterial load was observed during the 30-day storage. To overcome the above, l-cysteine-HCl that can function as both an oxygen scavenger and a nitrogen source for BB-12 cells was incorporated in the specific encapsulating mixtures: ACL-cys, ACNCL-cys, AML-cys. Thus, the viability of the BB-12 cells was enhanced up to 1.8–3.8%, coming in agreement with Sousa et al. [32] who observed improved storage stability of the same strain when l-cysteine-HCl was supplemented.

#### Survival Rates of Encapsulated BB-12 Under Frozen Storage (−18 °C)

Although frozen storage is widely applied for food preservation, it may have a negative impact on the viability of probiotic bacteria. The encapsulation of the probiotic strain BB-12 is expected to limit the damage commonly occurring during the freezing stage (freezing injuries) as well as during the entire storage period. The results presented in Fig. 2 indicate that the encapsulation of BB-12 in blends of alginate with other encapsulating agents significantly enhanced (*p* < 0.05) the survival of the probiotic cells during frozen storage compared to those encapsulated in plain alginate beads (A, AI). In all cases, significant viability loss of 3.1–6.2 log cfu g^−1^ occurred during the first days of storage due to the sudden exposure of the BB-12 cells to the injurious low temperature. The formation of ice crystals that provokes damage to the membrane structure of the probiotic cells and, thus, changing their physiological state can lead to cells’ death [33]. In the current study, the reinforcement of the alginate beads with polymer materials such as xanthan gum (AX) and κ-carrageenan (AC) increased the survival of the encapsulated cells by 41.0–52.1% (Fig. 2b, e). Τhe combination of alginate with whey and pectin (AWP, AWPI) led to survival rates of 48.7–49.2% at the end of the 30-day storage. Similarly, the addition of CNC (ACNC) in the alginate blend aided the maintenance of the viability of the BB-12 cells at a percentage of 49.6–52.1% (Fig. 2c, f). Moreover, the new approach of water replacement with milk for the production of alginate beads (AM) led to enhanced viability of up to 55.2% survival rates by the end of the storage (Fig. 2g). All milk-based beads (AM, AMI, AML-cys) retained their bacterial load above the required minimum of 6 log cfu g^−1^ for up to 20 days of storage. As in the case of refrigerated storage, whey-pectin, carrageenan, CNC, and milk provided the highest protection of BB-12 viability during frozen storage as well. In the case of frozen storage, in particular, greater survival rates (57.1%) were achieved when the cryoprotectant glycerol (AGl) was included into the encapsulating mixture (Fig. 2d), in comparison to other water-based encapsulating blends. Sultana et al. [34] also found a 100-fold higher cells’ survival when glycerol was incorporated into the alginate beads compared to alginate only or to the alginate-starch blend.

The addition of inulin in the alginate beads also improved the viability of BB-12 (*p* < 0.05) during frozen storage by increasing the survival by 0.6–24.4%. Raddatz et al. [19] also found that inulin had a protective effect on the probiotic cells during storage at –18 °C. Moreover, the beads containing l-cysteine-HCl demonstrated slightly increased stability during storage at –18 °C. Optimal results were achieved through the combination of milk, alginate, inulin and l-cysteine-HCl (AML-cys), as even after 30 days of storage the bacterial load was maintained at 6.1 log cfu g^−1^. Similar results are reported by Sousa et al. [32] who also observed improved behavior of the alginate beads during storage at –18 °C when l-cysteine-HCl was incorporated.

### Probiotics’ Survival During In Vitro Simulation of the GI Tract

**Fig. 3:**
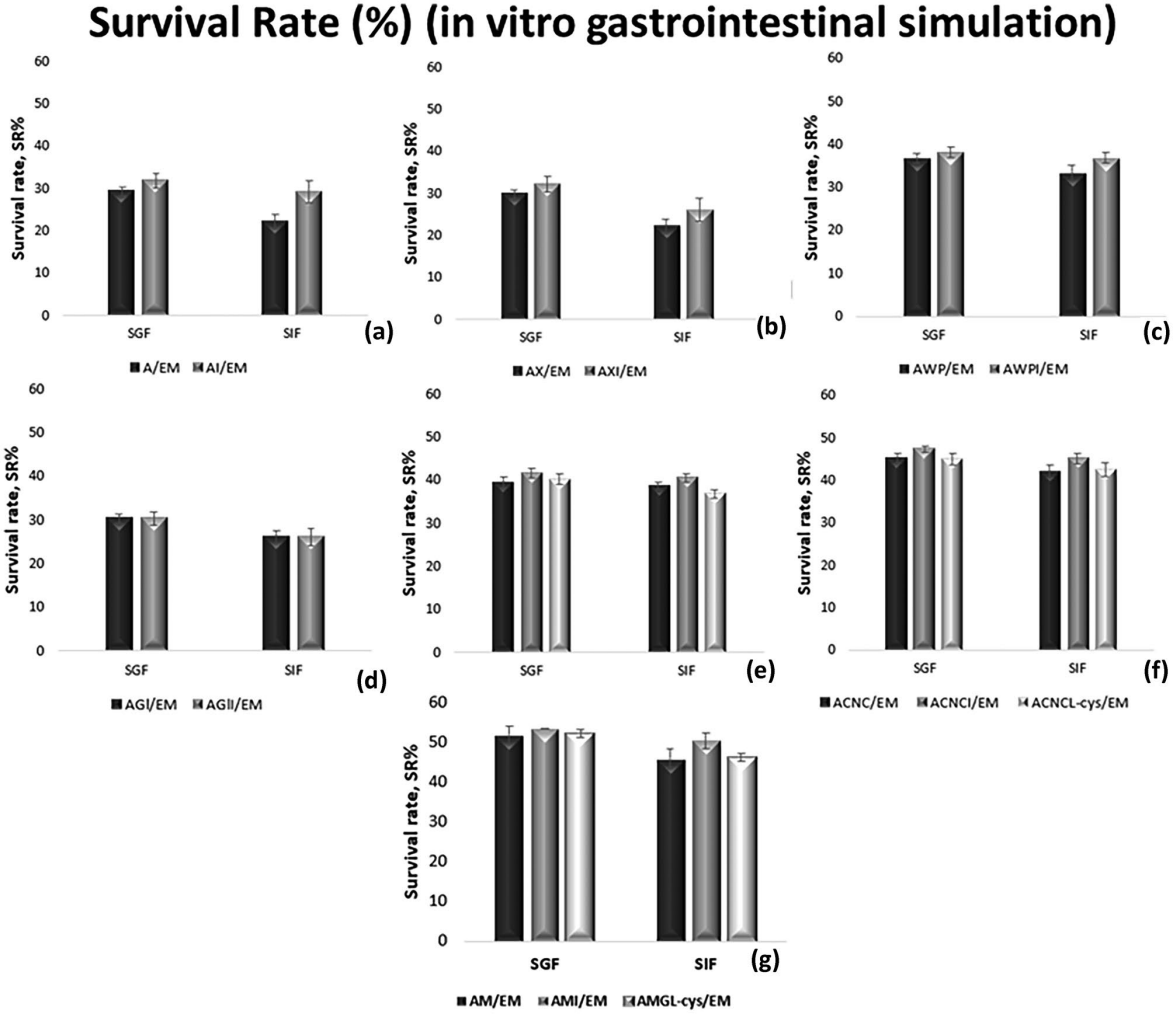
Survival rates of BB-12 after exposure to SGF and SIF conditions. * Values shown are means ± standard deviations (*n* = 3). ** Sample codes: A/EM: alginate, AI/EM: alginate-inulin, AX/EM: alginate-xanthan, AXI/EM: alginate-xanthan-inulin, AWP/EM: alginate-whey-pectin, AWPI/EM: alginate-whey-pectin-inulin, AGl/EM: alginate-glycerol, AGlI/EM: alginate-glycerol-inulin, AC/EM: alginate-carrageenan, ACI/EM: alginate-carrageenan-inulin, ACL-cys/EM: alginate-carrageenan-l-cysteine-HCl, ACNC/EM: alginate-CNC, ACNCI/EM: alginate-CNC-inulin, ACNCL-cys/EM: alginate-CNC-l-cysteine-HCl, AM/EM: alginate-milk, AMI/EM: alginate-milk-inulin, AML-cys/EM: alginate-milk-l-cysteine-HCl

For the assessment of the coating materials’ efficacy, the encapsulated cells were further exposed to in vitro simulated gastrointestinal conditions as described in “Survival of Encapsulated BB-12 Under simulated Gastrointestinal Conditions” subsection. Figure 3 shows survival of encapsulated BB-12 cells after this treatment. Reduction of the BB-12 populations was observed in all cases of encapsulating blends; however, the protection provided through encapsulation varied depending on the blend used (*p* < 0.05). The alginate, alginate-xanthan gum, and alginate-glycerol beads with (AI, AXI, AGlI) or without incorporated inulin (A, AX, AGl) presented the lowest bacterial loads, with survival rates of 22.3–29.3% at the end of the in vitro GI simulation. The addition of xanthan gum or glycerol in the mixture did not significantly affect the viability of BB-12 during the in vitro simulation. Similar results regarding the protective effect of glycerol in probiotic bacteria under GI conditions were also found by Sultana et al. [34]. On the other hand, the combination of sodium alginate with specific polymers, such as carrageenan (AC, ACI), CNC (ACNC, ACNCI) or whey and pectin (AWP, AWPI), may lead to the development of stronger, thicker, and more rigid beads, thus limiting the diffusion rate of the gastric acids and providing survival rates up to 42.0%. Moreover, the utilization of milk for the development of alginate-milk beads (AM) leads to increased survival of BB-12 cells during GI simulation, probably due to its complex composition that creates a favorable and protective environment for BB-12 cells (survival rates up to 50.3%). The combination of different materials has, also, been attempted by other researchers to confer improved protection to probiotic bacteria. For example, Pankasemsuk et al. [22] encapsulated the probiotic strain *L. casei* 01, through emulsification, in alginate-starch blends and observed that the higher the percentage of incorporated starch into the alginate beads, the greater its survival under simulated GI conditions. The protective effect of the alginate-starch blend was also observed by Sabikhi et al. [35] who examined the survival of *Lactobacillus acidophilus* at different concentrations of bile salts (1%, 1.5%, and 2%). Gerez et al. [36] encapsulated the strain *Lactobacillus rhamnosus* CRL 1505 in pectin or whey protein-pectin beads through emulsification and coated the occurring particles with whey protein for enhanced protection, achieving significantly higher survival rates of the encapsulated cells than the free cells when exposed to simulated GI conditions. Moreover, Zou et al. [37] reported an increase of 0.5 log cfu g^−1^ in the survival of *B. bifidum* F-35 by further addition of pectin in the alginate beads. However, no significant improvement was observed when starch was added in the alginate mixture. Furthermore, the incorporation of inulin into the encapsulating blends significantly enhanced (*p* < 0.05) the viability and increased the survival rates by up to 7.0%. Our results are in agreement with other researchers that reported the beneficial effect of inulin under GI conditions [26, 30, 31]. On the other hand, the addition of l-cysteine-HCl in the encapsulating blends provided only a slight increase of the survival rates (0.4–0.7%) when exposed to simulated GI conditions.

### Scanning Electron Microscopy Analysis of the Produced Beads

**Fig. 4:**
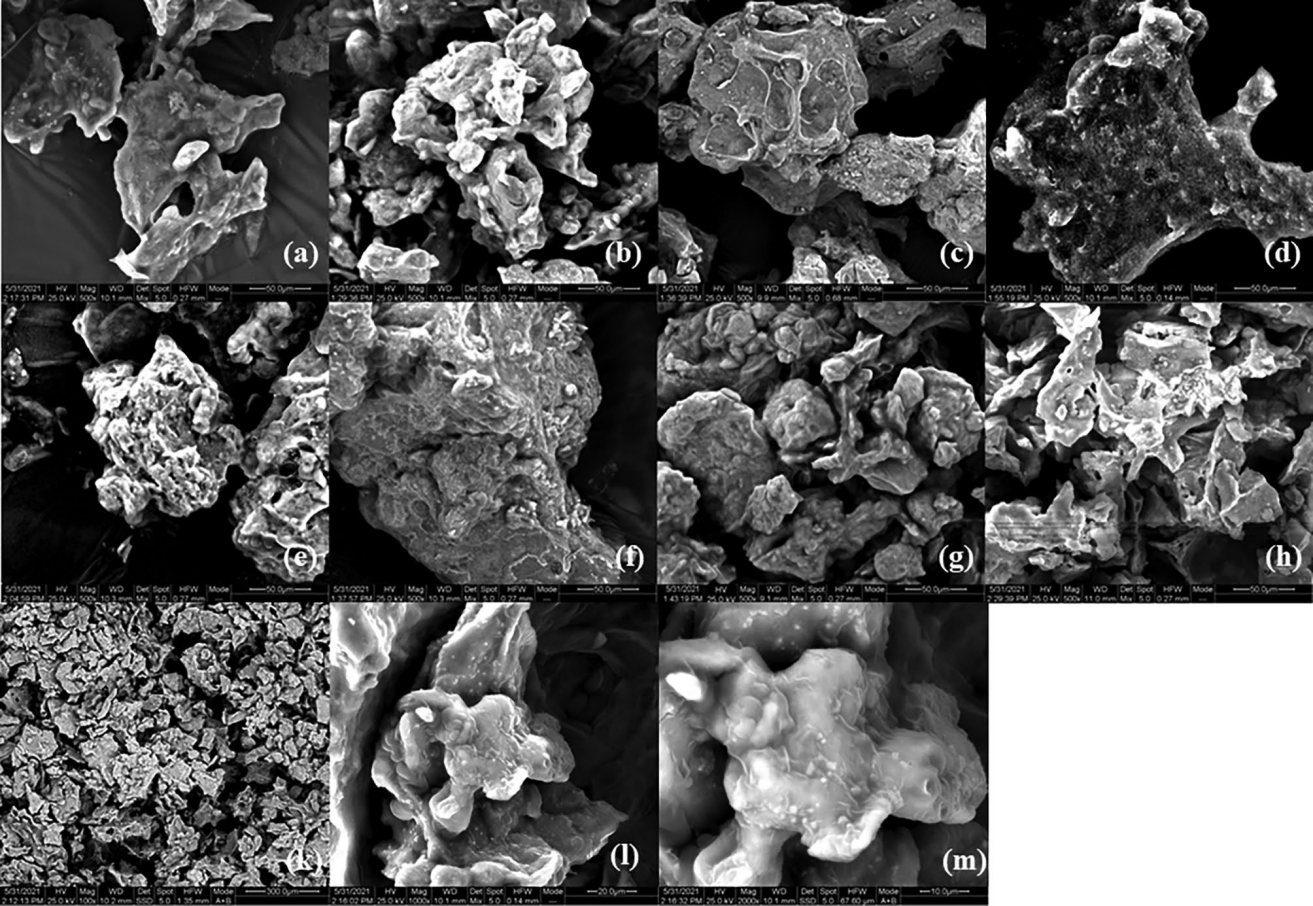
Scanning electron microscopy (SEM) images showing the overall structure of the produced beads with encapsulated BB-12. This figure shows freeze-dried formulations under 500 × magnification of **a** A, **b** AI, **c** AWP, **d** AX, **e** AGl, **f** AC, **g** ACNC, **h** AM, and freeze-dried formulations of sample Α under three magnifications: **k** 100 × , **l** 1000 × , and **m** 2000 × . * Sample codes: A/EM: alginate, AI/EM: alginate-inulin, AX/EM: alginate-xanthan, AXI/EM: alginate-xanthan-inulin, AWP/EM: alginate-whey-pectin, AWPI/EM: alginate-whey-pectin-inulin, AGl/EM: alginate-glycerol, AGlI/EM: alginate-glycerol-inulin, AC/EM: alginate-carrageenan, ACI/EM: alginate-carrageenan-inulin, ACL-cys/EM: alginate-carrageenan-l-cysteine-HCl, ACNC/EM: alginate-CNC, ACNCI/EM: alginate-CNC-inulin, ACNCL-cys/EM: alginate-CNC-l-cysteine-HCl, AM/EM: alginate-milk, AMI/EM: alginate-milk-inulin, AML-cys/EM: alginate-milk-l-cysteine-HCl

The scanning electron microscopy (SEM) analysis micrographs for the beads produced with different encapsulating blends are presented in Fig. 4. The surface of the beads was examined at the same magnification of 500 × (Fig. 4) for comparison reasons.

However, for the needs of the SEM analysis, the beads were first subjected to freeze drying. This resulted to samples with irregular shape and size. Thus, the initially soft and smooth surface of beads turned into a rough one with irregular concavities and wrinkles, due to the removal of water from the hydrogel. This sponge-like external structure occurs due to the fast sublimation of the frozen water from the beads, leading to pores formed in the place of the ice crystals [38]. According to Fig. 4, AI and AGl samples have similar surface characteristics (Fig. 4b, e). AC and AWP samples exhibit a more compact structure with a less porous surface (Fig. 4c, f), whereas the ACNC sample is characterized by a spongier structure (Fig. 4g). Moreover, the structure of A and AM samples is quite similar, with less concavities (Fig. 4a, f).

In order to provide a more detailed approach of samples’ microstructure, a randomly chosen sample is presented in Fig. 4k, l, m under different magnifications (100 × , 1000 × , and 2000 ×). The lack of homogeneity, regarding the size and shape of the beads, is clearly captured in Fig. 4k. Beads of various sizes and shapes are dispersed, whereas clusters of beads have been created. This formation can be attributed to the cohesive nature of the encapsulating agents used [39]. It must be noted that Fig. 4k indicates the absence of free bacteria, thus confirming the successful encapsulation of BB-12 cells.

## Conclusions

The encapsulation of BB-12 cells through emulsification, in most cases, improved the survival of the BB-12 cells both during storage or transit through the GI tract. Alginate on its own was not efficient in maintaining probiotics’ viability. On the other hand, its combination with certain conventional (carrageenan) or novel (CNC) materials enhanced the protective properties of the occurring beads. The best results were provided when water was replaced by milk during the encapsulation process (AM). Interestingly, these materials, due to the dense structure of the beads produced, were effective not only in protecting BB-12 at low storage temperatures (4 °C and −18 °C) but also during the in vitro simulation of the GI tract. Consequently, the emulsification with the use of encapsulating blends proposed in this study may significantly maintain the viability of probiotic bacteria during both storage and simulated GI conditions in a simple and cost-effective manner. The proposed encapsulation systems can be studied for the enrichment of food products, as they are promising protective matrices for BB-12 cells. Thus, they will be probably able to provide viability enhancement of BB-12 cells during food manufacturing and storage, as well as during simulated GI conditions.

## Data Availability

All data generated or analyzed during this study are included in this published article.
